# The Essential Oil of *Thymbra capitata* and its Application as A Biocide on Stone and Derived Surfaces

**DOI:** 10.3390/plants8090300

**Published:** 2019-08-24

**Authors:** Rossella Gagliano Candela, Filippo Maggi, Giuseppe Lazzara, Sergio Rosselli, Maurizio Bruno

**Affiliations:** 1 Dipartimento di Scienze e Tecnologie Biologiche, Chimiche e Farmaceutiche (STEBICEF), Università degli Studi di Palermo, Viale delle Scienze, I-90128 Palermo, Italy; 2 School of Pharmacy, University of Camerino, Via Sant’Agostino 1, I-62032 Camerino, Italy; 3 Dipartimento di Fisica e Chimica “Emilio Segrè”, Università degli Studi di Palermo, Viale delle Scienze, pad. 17, I-90128 Palermo, Italy and Consorzio Interuniversitario Nazionale per la Scienza e Tecnologia dei Materiali, INSTM, Via G. Giusti, 9, I-50121 Firenze, Italy

**Keywords:** *Thymbra capitata*, biological inhibition, essential oil, Pickering emulsion, stone surfaces, biodeteriogens, natural biocide, cultural heritage

## Abstract

Many chemicals used nowadays for the preservation of cultural heritage pose a risk to both human health and the environment. Thus, it is desirable to find new and eco-friendly biocides that can replace the synthetic ones. In this regard, plant essential oils represent effective alternatives to synthetic substances for the preservation of historical monuments. *Thymbra capitata* (syn. *Thymus capitatus*) is a medicinal and aromatic plant growing in the Mediterranean area and endowed with important pharmacological properties related to its essential oil. Among them, the antimicrobial ones make the *T. capitata* essential oil an ideal candidate for industrial applications; for instance, as biocide for the inhibition and elimination of biological patinas of cyanobacteria and green algae on historical monuments. In the present work, we studied the chemical composition of the essential oil from *T. capitata* growing in Malta by gas chromatography-mass spectrometry (GC/MS). The major volatile component is the phenolic monoterpene carvacrol (73.2%), which is capable of damaging the cytoplasmic membrane and to interfere both in the growth curve and in the invasive capacity, though the contribution of minor components γ-terpinene and *p*-cymene cannot be disregarded. For the oil application on the stone surface, Pickering emulsions systems were prepared with an essential oil/water 1:3 mass ratio stabilized with kaolinite at 4 mass% in the presence of Laponite^®^; this allowed to limit the fast volatility of the oil and guaranteed a better application and an easier removal from the artefacts attacked by biodeteriogens both indoor and outdoor. This formulation caused the elimination of biodeteriogens from treated surfaces without residuals or films on artworks surface, and the effect was retained up to four months.

## 1. Introduction

The biological properties of the plant species of the genera *Thymus*, *Thymbra*, and *Satureja* were known from the ancient times by the Egyptians, Greeks, and Romans [1,2,3]. These civilities used the plant and their essential oils for many health problems and therapies. Plinio The Old (I century B.C) knew the properties of their essential oils and wrote different chapters in the historical book “Naturalis Historia”. The above genera are part of the complex Lamiaceae family [4,5,6] and are particularly prolific of chemotypes that rise from the adaptation to various environmental conditions. The majority of the good biological proprieties of the *Thymus* and *Thymbra* ssp. are related to the presence of polyphenol derivatives in their extracts and infusions [7]. On the other hand, the essential oils have several applications in different sectors as preservative, antioxidant, flavouring, and so on [8,9,10,11,12,13,14,15,16]. The compositions of these essential oils are characterized by a high percentage of monoterpenoids (around 90%). Carvacrol and thymol are very frequent, accompanied by the couple *p*-cymene/γ-terpinene [17,18]. Other important components occurring in minor quantity are linalool, borneol, and 1,8-cineole [19,20]. Different chemotypes of the above genera are used as spices in traditional foods in many parts of the world because of their good smell and taste and their antimicrobial, antifungal, and antioxidant activities. Consequently, they have the ability to inhibit the growth of a wide range of pathogenic microorganisms [21,22,23,24,25,26,27]. In particular, the antimicrobial properties of the essential oils of *Thymbra capitata* (L.) Cav. (formerly *Thymus capitatus* (L.) Hoffmanns. & Link) and its various components have been well documented [28,29] and the chemical composition of several accessions published in several papers [25,28,30,31,32,33,34,35,36,37,38,39,40,41]. The main mode of action of this oil seems to be the interaction with cell membrane causing alteration of lipid bilayer. Further, among its main constituents, carvacrol is reported as the most active although a synergist effect may be given by pure hydrocarbons such as *p*-cymene and γ-terpinene, which are able to cause membrane swelling facilitating the penetration of carvacrol into the cell. Structure–activity relationships (SAR) studies evidenced that hydrophobicity together with the presence of a free hydroxyl group and a delocalized system are a key factor in the antimicrobial activity of carvacrol. Indeed, the capacity to form hydrogen bond and to release protons are at the basis of microbial cell death through conformational modifications of the cell membrane [42].

Many heterotrophic microorganisms interact with historical materials such as the stone, the architectonic surfaces, and stone derivatives (ceramic). Stucco, mortars, fresco, and stones of the statues are good growing media because of their porosity, condensation, capillary rise, lack of ventilation, humidity, and the heat due to the sunshine. Their mineralogical composition makes them very bio-selective. The microorganisms can cause different degradations and alterations of the artefact surfaces. They create the biological substrate on the surface and left pigments that stain and spot the artwork. Furthermore, the biological activity of microorganisms can produce acids that are able to corrode and modify the surface. Noteworthy, the presence of one species can favour the colonization of other species. Generally, the stone artefacts (historical monuments, ancient buildings, frescos, fountains, statues, and so on) are affected by several biodeteriogens as fungi, green algae, cyanobacterias, and lichens, depending on the environment, indoor or outdoor (museums, churches and crypts, hypogeums, gardens, urban environments, coastal areas, fountains and nymphs, arcades, and so on) [43]. Fungi, algae, and bacteria cause the loss of material and surface detachment because of the hyphae capacity to penetrate in the substrate for 10 mm in the stone and much more in the mortar [44,45]. Several bacteria such as *Streptomyces, Micromonospora,* and *Nocardia* produce a biological white film on the surface like a saline efflorescence. Green algae and cyanobacteria alter the original surface of the artwork and, in addition to aesthetic disfigurement, cause physico-chemical degradation.

Recently, also in the restore sector, the interest in biocompatible and eco-friendly solutions has increased. Many chemical products (quaternary ammonium compounds, chlorides, hypochlorites, and so on) are not used anymore and, when possible, a “green solution” is preferred [44,46,47,48,49,50]. There are less publications and application of natural eco-biocide for the stone artworks, but if these artefacts are outdoor, they are more steadily and repeatedly attacked by biodeteriogens [45,51,52]. In this work project, we present the study of the essential oil of *T. capitata* collected in Malta and how we used it for the biochemical inhibition of microorganisms, such as cyanobacterias and green algae, colonizing outdoor stone surfaces. The goal was to create, by means of a Pickering emulsion, an inorganic matrix containing the essential oil of *T. capitata*, easily applicable and removable from stone surfaces. The emulsion with the essential oil must ensure the elimination of biodeteriogenic attacks in an ecological way, leaving no residue, respecting the stone surface and the environment, and ensuring a biocidal action over time. Essential oils have been used in the cultural assets field for the same purposes, so far, but using organic products such as agar-agar and funori as application matrix (gelling polysaccharides that have good application, but cost as much as essential oils) [51]. The method we present here has a simple application even in vertical surfaces, with a low cost. Moreover, the essential oil of *T. capitata* showed a very interesting biodeteriogenic action when compared with other essential oils of *Thymus* spp.

## 2. Results and Discussion

### 2.1. Essential Oil

The chemical composition of the essential oil from *T. capitata* is reported in Table 1. A total of 28 components were identified by GC/MS, accounting for 99.9% of the total oil. The essential oil composition was characterized by monoterpeneoids (96.7%), with oxygen-containing compounds (78.9%) being the predominant compounds followed by monoterpene hydrocarbons (17.8%). Sesquiterpenes were present in little amounts (2.6%). The major component was the phenolic monoterpene carvacrol (73.2%), followed by its biogenetic precursors γ-terpinene (6.9%) and *p*-cymene (4.3%). Other components occurring in percentages >1 were linalool (2.6%), (*E*)-caryophyllene (2.6%), α-terpinene (1.4%), α-thujene (1.4%), borneol (1.4%), and myrcene (1.1%).

The essential oil chemical profile herein described for a wild population growing in Malta resulted consistent with that previously reported by Merino et al. [53] for a Spanish accession. Indeed, the latter was characterized by carvacrol (73.8%), *p*-cymene (9.2%), and γ-terpinene (5.3%).

The biocidal properties on cultural heritage artworks of the essential oils from the genus *Thymus* have been described in other works, and very good results have been obtained for *Thymus vulgaris* L., *Thymus serpyllum* L., and *Thymus pulegioides* L. The oils of these species were shown to be active against fungi (e.g., *Alternaria alternata, Aspergillus niger, A. versicolor, Cladosporium sphaerospermum, Penicillium chrysogenum, P. simplicissimum, Rhizopus stolonifer*), Chlorophylleae Algaes (e.g., *Chlorella ellipsoidea, Apatococcus lobatus*), and cyanobacterias (*Nostoc* spp.*, Phormidium* spp.*, Gloeocapsa compacta, G. rupestris, G. sanguinea, Chroococcus lithophilus*) [54,55,56].

Generally speaking, the main problems in the usage of essential oils on outdoor stone surfaces are attributable to their high volatility, low persistence, and difficult application. Consequently, we decided to obtain a physic support suitable for a correct use by preparing Pickering emulsion systems. 

### 2.2. Pickering Emulsion

Pickering emulsions are used in the industrial fields, but, recently, their application in other sectors has drastically increased. Pickering emulsions are valid alternatives to surfactants for the formation of an emulsion. The emulsions are made up of fine particles that form a shell around the oil drop or water drop [57]. Water-in-oil (W/O) emulsions are widely used for different “soft” matter applications: pharmaceutical, food, personal care, agriculture and, as described in the present paper, in the restoration of cultural heritage [58,59,60]. Today, the use of Pickering particles as stabilizers for emulsions has received attention owing to their ability to adsorb irreversibly at the liquid/liquid interface [61,62]. Pickering stabilization happens when particles assemble at the water–oil (W/O) interface making a mechanical (steric) barrier that protects the emulsion droplets against coalescence [63]. A correct Pickering stabilization foresees that the dimensions of the adsorbed particles are at least an order of magnitude smaller than the emulsion droplet size [64,65]; smaller particles will give a higher grouping and more homogeneous layer at the interface preventing coalescence. Particles size is also directly proportional to desorption free energy as it can be calculated by the following formula:

ΔG_d_ = πr^2^γ_ow_(1 ± |cos(θ)|)^2^,

where r is the particle radius, γ_ow_ is the interfacial tension between oil and water phases, and θ is the three phases contact angle.

In this work, for an application on the stone surfaces, several Pickering emulsions were prepared by mixing the *T. capitata* essential oil and water, in a ratio of 1:3, with different clays: kaolinite (Kao), sepiolite (Sep), and Laponite^®^ (Lap), in a wide concentration range. Nanoclays were considered because they are promising tools in cultural heritage conservation protocols as demonstrated in recent works [66,67].

**Figure 1:**
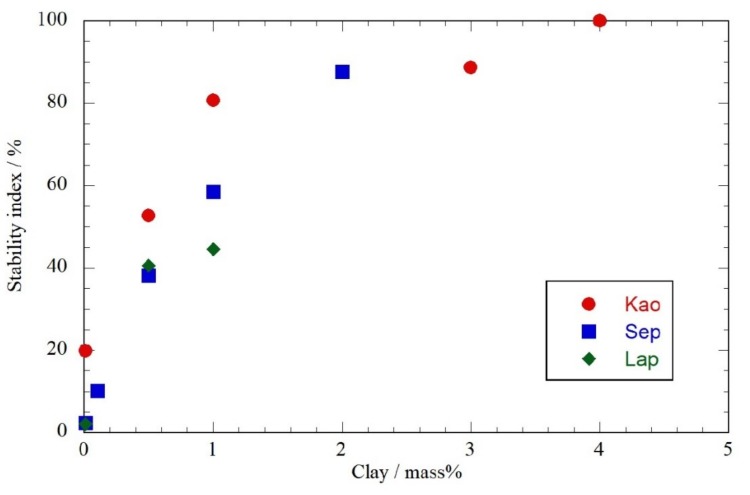
Pickering emulsion stability index as functions of clay content.

Figure 1 reports the stability index obtained for the Pickering emulsions based on the three different clays as functions of clay concentration. It should be noted that Lap above 1 mass% did not allow us to obtain a homogeneous dispersion and, therefore, a limit in the stabilizing ability of this nanoclay was evidenced. On the other hand, the most effective clay in oil stabilization was Kao that at 4 mass% allowed total stabilization of the oil. Sep clay can be considered a good dispersing agent, although its efficacy could not exceed 85% of oil stabilization.

**Figure 2:**
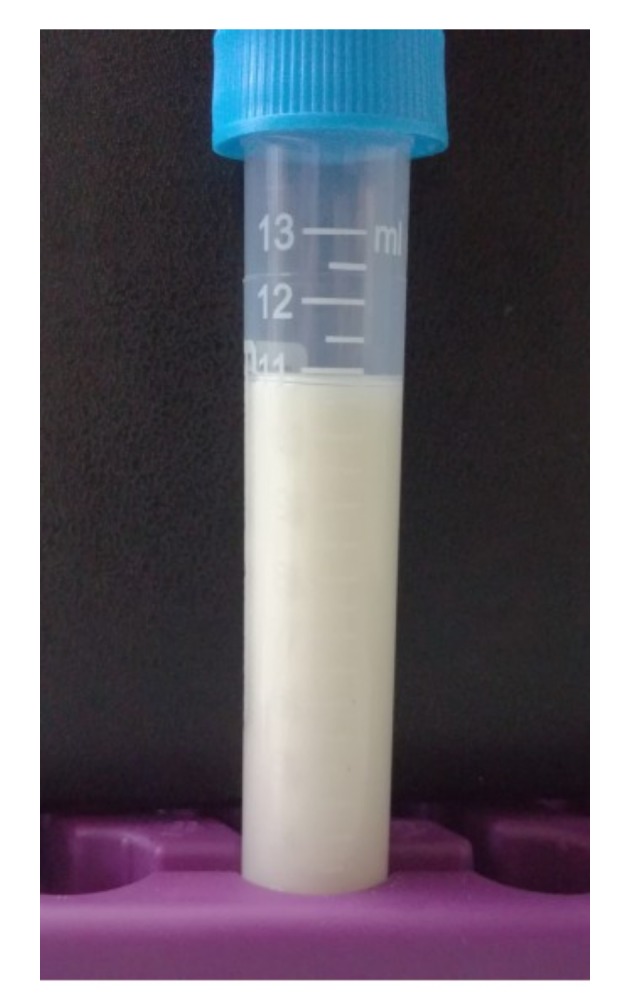
Optical photo of oil in water Pickering emulsion with 4 mass% of Kao in the presence of Laponite^®^ as thickener agent.

On the basis of these results, we considered a Pickering emulsion with an essential oil/water 1:3 mass ratio stabilized with Kao at 4 mass% to which, based on gel formation ability of laponite^®^ clay [68], the latter was added as thickener agent (4%). A milk-like gel dispersion was obtained, which appeared suitable for applications in surface cleaning (Figure 2).

### 2.3. Application

**Figure 3:**
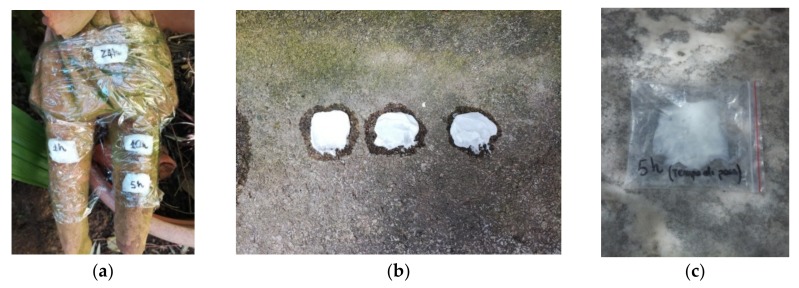
Application of Pickering emulsion on the surfaces: (**a**) ceramic, (**b**) cement grit, and (**c**) marble.

The chosen emulsion was tested (30 March 2019, temperature 14°C, humidity 64%) on three outdoor surfaces (ceramic, marble, cement grit), attacked by biological agents (green algae Clorophyceae and cyanobacteria) by applying it on their surfaces and covering with a polyethylene film, for some hours (Figure 3).

For ceramic surface (Figure 3a), the laying times were 1, 5, 10, and 24 h, after which the emulsion was removed with pads and water. Three tests were applied on the cement grit (1, 5, 24 h laying time) (Figure 3b). Then, the first one was removed by mechanical action, and the other two by washing with water. The emulsion was also tested on an outdoor marble surface subjected to atmospheric agents and presenting a biological attack (Figure 3c). In this case, the emulsion was left for 5 h and then removed with water and pads.

**Figure 4:**
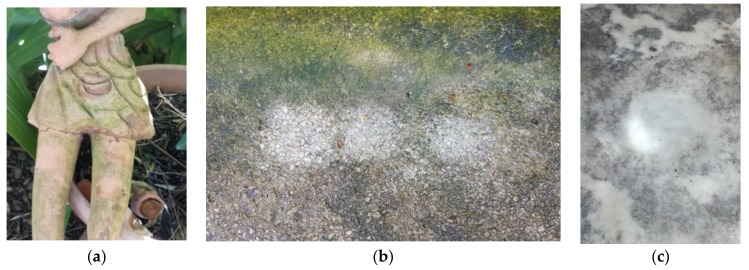
Surfaces after the application of Pickering emulsion: (**a**) ceramic, (**b**) cement grit, and (**c**) marble.

The results were very satisfying for all the tested materials, as shown in Figure 4, and the treated surfaces looked clean and devoid of biodeteriogens. After four months, the surfaces are still clean and free of the biodeteriogens.

## 3. Materials and Methods

### 3.1. Plant Material

Aerial parts of *T. capitata* were collected at Paradise Bay, Malta (35°58’53” N, 14°19’58” E), in June 2018. Typical specimens (PAL 18/64), identified by Mr. Emanuele Schimmenti, were deposited in the Department STEBICEF, University of Palermo, Palermo, Italy.

### 3.2. Essential Oil Distillation

The fresh samples were ground in a Waring blender and then subjected to hydrodistillation for 3 h, according to the standard procedure previously described [69]. The oils were dried over anhydrous sodium sulphate and then stored in sealed vials, at −20 °C, ready for the gas chromatography-mass spectrometry (GC/MS) analysis. The sample yielded 0.35% (*w/w*) of oil.

### 3.3. Qualitative and Quantitative Analyses of Essential Oil (GS and GC/MS)

GC/MS analysis was performed on an Agilent Technologies 6850 N gas chromatograph coupled to a mass spectrometer (Agilent Technologies 5973) using a HP-5 MS (5% phenylmethylpolysiloxane, 30 m, 0.25 mm i.d., 0.25 μm film thickness; J & W Scientific, Folsom) capillary column. The oven temperature programme was as follows: 5 min at 60 °C, subsequently 4 °C/min up to 220 °C, then 11 °C/min up to 280 °C, held for 15 min, for a total run of 67.29 min. Injector and detector temperatures were 280 and 300 °C, respectively. Helium (He) was used as the carrier gas, at a flow rate of 1 mL/min. Split ratio, 1:50; acquisition mass range, *m/z* 29–400. All mass spectra were acquired in electron-impact (EI) mode with an ionization voltage of 70 eV. Oil samples were diluted to 1:100 in *n*-hexane, and the volume injected was 2 μL. For 23 out of 28 essential oil constituents (Table 1), the identification consisted in the comparison of peak retention time, retention index, and mass spectrum with that of authentic standards (Sigma-Aldrich, Milan, Italy). Otherwise, the peak identification was based on the combination of linear retention indices and mass spectra with those reported in the WILEY275, NIST 17, ADAMS, and FFNSC2 libraries. Quantification of essential oil components was performed by peak-area normalization by considering an equal response factor for the different chemical classes. 

### 3.4. Pickering Emulsion

In this work, Pickering emulsion was used as support to enable *T. capitata* essential oil application on stones surfaces. Water, oil, and clays were mixed to make the emulsion. For all Pickering emulsions, the mass percentage of clays was systematically changed, while the oil/water ratio was fixed at 1:3. A literature protocol was used to quantify the emulsion stability [59]. Briefly, the emulsions were sonicated (Transsonic 310/h) for 5 min, and then centrifuged at 1000 rpm (Heraeus Multifuge 3S+ centrifuge Thermo scientific). The quantity of separated oil phase was determined from optical photos after proper calibrations using ImageJ software. The stability index was calculated in terms of oil percentage incorporated into the emulsion and that did not show separation after the centrifugation protocol. 

## 4. Conclusions

The Pickering emulsion of the essential oil of *T. capitata* is a typical emulsion that is called “lean emulsion” in the field of restoration of cultural assets. This emulsion is characterized by the presence of two immiscible products: an apolar substance (oil or mix of hydrocarbons) and a more polar one (water). Usually, a surfactant is added to emulsify these products together. In our case, the Pickering emulsion was characterized by the absence of surfactants and by the presence of a clay that acts as an emulsifier (kaolin) and another gelling agent (laponite). When the *T. capitata* oil Pickering emulsion is applied to a biologically degraded stone surface, an interaction is created between water, oil, stone surface, algae or bacteria. The biocidal phenolic components of the oil (carvacrol, thymol) act on and damage the plasma membrane of the cells, leading to biodeteriogens’ death. Furthermore, this emulsion shows a cleaning power, thanks to the presence of water. Once the emulsion has been removed, dead biodeteriogens are also detached. Months later, it was observed that the biocidal action of the essential oil of *T. capitata* is still ongoing. The parts treated with the emulsion are still free of biological degradation and optically clean. There are currently no contraindications to the use of this essential oil alone or encapsulated in emulsions and polysaccharides. These are totally natural products that intervene microscopically at the cellular level, and, unlike the current quaternary ammonium salt chemicals, are not dangerous for the environment and the operator. The use of natural and ecological products in the field of restoration is growing rapidly and this product is harmless to the cultural heritage, to the operator and to the general environment. The only downside, if it is, is the typical smell of the oil, which persists for several days.

## Figures and Tables

**Table 1:** Essential oil composition of *Thymbra capitata*.

No	Component ^a^	RI ^b^	RI Lit. ^c^	% ^d^	ID ^e^
1	α-thujene	921	924	1.4 ± 0.3	RI,MS
2	α-pinene	929	932	1.0 ± 0.2	Std,RI,MS
3	camphene	936	946	0.5 ± 0.1	Std,RI,MS
4	sabinene	966	969	0.1 ± 0.0	Std,RI,MS
5	β-pinene	971	974	0.1 ± 0.0	Std,RI,MS
6	1-octen-3-ol	973	974	0.4 ± 0.1	Std,RI,MS
7	myrcene	985	988	1.1 ± 0.2	Std,RI,MS
8	3-octanol	994	988	0.2 ± 0.0	RI,MS
9	α-phellandrene	1001	1002	0.2 ± 0.0	Std,RI,MS
10	δ-3-carene	1005	1008	Tr ^f^	Std,RI,MS
11	α-terpinene	1012	1014	1.4 ± 0.3	Std,RI,MS
12	*p*-cymene	1019	1020	4.3 ± 0.7	Std,RI,MS
13	limonene	1023	1024	0.7 ± 0.2	Std,RI,MS
14	γ-terpinene	1052	1054	6.9 ± 1.3	Std,RI,MS
15	*cis*-sabinene hydrate	1062	1065	0.4 ± 0.1	RI,MS
16	terpinolene	1082	1086	0.1 ± 0.0	Std,RI,MS
17	*trans*-sabinene hydrate	1094	1098	0.1 ± 0.0	RI,MS
18	linalool	1098	1095	2.6 ± 0.5	Std,RI,MS
19	borneol	1161	1165	1.4 ± 0.3	Std,RI,MS
20	terpinen-4-ol	1171	1174	0.4 ± 0.1	Std,RI,MS
21	α -terpineol	1186	1186	0.1 ± 0.0	Std,RI,MS
22	carvone	1238	1239	0.2 ± 0.0	Std,RI,MS
23	thymol	1293	1289	0.2 ± 0.1	Std,RI,MS
24	carvacrol	1301	1298	73.2 ± 3.1	Std,RI,MS
25	carvacrol acetate	1368	1370	0.4 ± 0.2	RI,MS
26	(*E*)-caryophyllene	1414	1417	2.6 ± 0.4	Std,RI,MS
27	α-humulene	1447	1452	Tr	Std,RI,MS
28	caryophyllene oxide	1573	1583	0.1 ± 0.0	Std,RI,MS
	Total identified (%)			99.9 ± 0.1	
	Grouped compounds (%)				
	Monoterpene hydrocarbons			17.8	
	Oxygenated monoterpenes			78.9	
	Sesquiterpene hydrocarbons			2.6	
	Oxygenated sesquiterpenes			Tr	
	Aliphatics			0.6	

^a^ Components are listed according to their elution from a HP-5MS column. ^b^ Linear retention index (RI) experimentally calculated using the Van den Dool and Kratz formula using a mixture of *n*-alkanes (C_8_–C_24_). ^c^ Literature RI taken from NIST 17 or ADAMS libraries. ^d^ Relative peak area percentage as mean of three measurements ± standard deviation. ^e^ Peak assignment method: RI, correspondence of calculated value with those stored in NIST 17 or ADAMS libraries; MS, similarity of mass fragmentation with those recorded in NIST 17, WILEY 275, FFNSC2, and ADAMS libraries; Std, comparison with available analytical standard (Sigma-Aldrich, Milan, Italy). ^f^ Tr, traces, % < 0.05.
